# Crystals of TELSAM–target protein fusions that exhibit minimal crystal contacts and lack direct inter-TELSAM contacts

**DOI:** 10.1098/rsob.210271

**Published:** 2022-03-02

**Authors:** Supeshala Nawarathnage, Sara Soleimani, Moriah H. Mathis, Braydan D. Bezzant, Diana T. Ramírez, Parag Gajjar, Derick R. Bunn, Cameron Stewart, Tobin Smith, Maria J. Pedroza Romo, Seth Brown, Tzanko Doukov, James D. Moody

**Affiliations:** ^1^ Department of Chemistry and Biochemistry, Brigham Young University, Provo, UT, USA; ^2^ Department of Natural Sciences, California State University Chico, Chico, CA, USA; ^3^ Macromolecular Crystallography Group, Structural Molecular Biology Resource, Stanford Synchrotron Radiation Lightsource, Menlo Park, CA, USA

**Keywords:** TELSAM, polymer-forming protein crystallization chaperone, covalent protein crystallization chaperone, X-ray crystallography, protein crystallization method, protein polymer

## Abstract

While conducting pilot studies into the usefulness of fusion to TELSAM polymers as a potential protein crystallization strategy, we observed novel properties in crystals of two TELSAM–target protein fusions, as follows. (i) A TELSAM–target protein fusion can crystallize more rapidly and with greater propensity than the same target protein alone. (ii) TELSAM–target protein fusions can be crystallized at low protein concentrations. This unprecedented observation suggests a route to crystallize proteins that can only be produced in microgram amounts. (iii) The TELSAM polymers themselves need not directly contact one another in the crystal lattice in order to form well-diffracting crystals. This novel observation is important because it suggests that TELSAM may be able to crystallize target proteins too large to allow direct inter-polymer contacts. (iv) Flexible TELSAM–target protein linkers can allow target proteins to find productive binding modes against the TELSAM polymer. (v) TELSAM polymers can adjust their helical rise to allow fused target proteins to make productive crystal contacts. (vi). Fusion to TELSAM polymers can stabilize weak inter-target protein crystal contacts. We report features of these TELSAM–target protein crystal structures and outline future work needed to validate TELSAM as a crystallization chaperone and determine best practices for its use.

## Graphical abstract

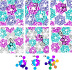

## 1. Background

Atomic-resolution protein structures are essential for structure–function studies, structure-based drug design and biomedical protein engineering. X-ray crystallography remains an important technique to determine atomic-level protein structure, especially of proteins too small for single-particle cryo-electron microscopy. X-ray crystallography additionally provides high-resolution protein structures that can be docked into lower resolution cryo-electron maps. Protein crystals are also needed for micro-electron diffraction [1] and time-resolved diffraction using X-ray free-electron lasers [2]. Current protein crystallization methods are successful for only about 10% of all known proteins [3] and constitute a lengthy, laborious and expensive process [4]. Lack of high-resolution structures hampers the structure–function studies of many proteins. There is a critical need for new protein crystallization methods that require less labour, time and resources and that can induce the crystallization of a wider range of proteins. Our ultimate goal is to develop a protein crystallization chaperone that consistently meets the following requirements: (i) is easy to express in *Escherichia coli* and purify with sufficient yield to screen crystallization conditions, even when fused to target proteins; (ii) enables fused targets to be sufficiently soluble for crystallization screens; (iii) crystallizes in no more than 30 days; (iv) forms crystals that diffract to better than 2 Å with an estimated mosaicity less than 2°; and (v) results in target proteins being well resolved in the crystallographic lattice following molecular replacement or direct phasing.

A pH-sensitive mutant of the polymer-forming sterile alpha motif (SAM) domain of human translocation ETS leukaemia (TEL) protein (TELSAM) was previously engineered [5]. It was then shown that genetic fusion of a panel of target proteins of interest to this TELSAM protein polymer could consistently achieve their crystallization [6,7]. While many of the resulting crystals were too disordered to permit structure determination, we propose that continued investigation into the requirements for obtaining well-ordered crystals of TELSAM–target protein fusions is warranted for the following reasons. (i) The long TELSAM polymers are expected to confer increased avidity to any weak crystal contacts made by fused target proteins, allowing crystallization of a greater fraction of proteins. (ii) The regular spacing of target proteins along the sixfold helical TELSAM polymer is expected to pre-programme much of the symmetry and spacing of the resulting crystal lattice. (iii) Fusion to TELSAM is expected to force target proteins to participate in the resulting crystal lattice, ordering the target proteins and allowing them to be resolved in electron density maps. (iv) The spacing between adjacent polymers can adjust to accommodate fused target proteins with a wide range of sizes. (v) Connections between the target protein and the TELSAM polymer need not be perfectly rigid because the resulting crystal lattice contacts are expected to provide the remainder of the needed rigidity, forcing the target protein to choose among a small number of low-energy orientations available to it.

**Figure 1.**
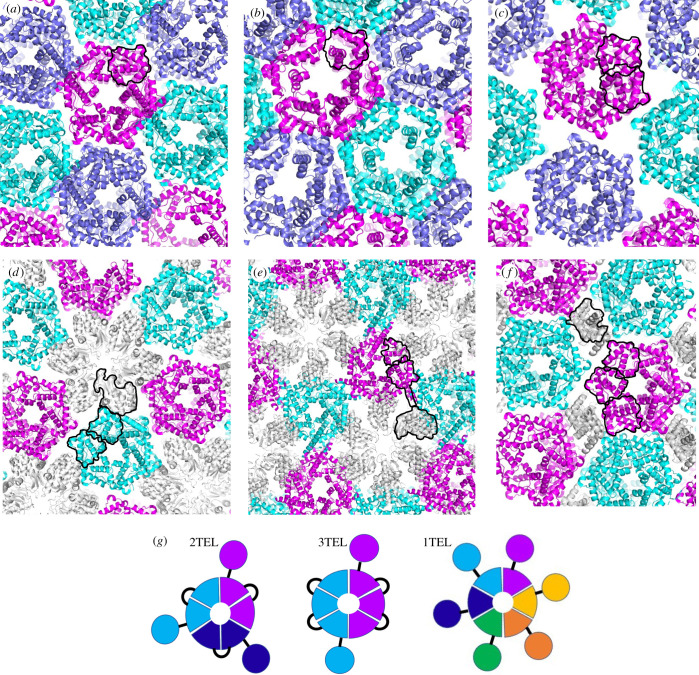
All currently reported structures involving TELSAM polymers feature direct inter-polymer crystal contacts. TELSAM polymers are shown in cartoon representation and coloured in magenta, cyan and violet. Fused target proteins are coloured grey. In each image, a single polypeptide has been indicated with black outlines around each of its constituent sub-domains. (*a*) PDB ID 1JI7: 1TEL alone [5], (*b*) PDB ID 1LKY: 1TEL E222R mutant [8], (*c*) PDB ID 2QB1: 2TEL alone [6], (*d*) PDB ID 2QB0: 2TEL–lysozyme fusion [6], (*e*) PDB ID 2QAR: 2TEL–helix–lysozyme fusion [6], (*f*) PDB ID 5L0P: 3TEL–ferric uptake regulator fusion [7], (*g*) schematic of 2TEL, 3TEL and 1TEL. Individual polypeptides are offset with unique colours. Circles denote target proteins, wedges denote TELSAM subunits and black lines denote linkers.

All crystal structures of TELSAM alone or genetically fused to target proteins reported to date feature direct inter-TELSAM polymer contacts (figure 1*a–f*) [5–8]. This observation led us and others [6] to hypothesize that strong inter-polymer contacts are essential to obtain well-diffracting crystals of TELSAM–target protein fusions. Previously reported TELSAM–target fusions used 2TEL (which fuses two copies of the SAM domain in tandem and thus displays three copies of the target protein around the sixfold TELSAM polymer axis) or 3TEL (which fuses three copies of the SAM domain in tandem and thus displays two copies of the target protein around the TELSAM polymer axis) [6,7] (figure 1*g*). Both of these architectures allowed direct inter-polymer contacts while also accommodating the target protein in the crystal lattice (figure 1*d–f*). We hypothesized that fusion of a target protein to 1TEL (which would display six copies of the target protein around the TELSAM polymer axis; figure 1*g*) might prevent TELSAM from forming any inter-polymer contacts. Instead, all crystal contacts would need to be made by the fused target protein. We further hypothesized that the inability to make direct inter-polymer contacts might preclude 1TEL–target fusions from forming well-diffracting crystals. We tested this hypothesis and found that 1TEL–target fusions can indeed form diffraction-quality crystals that permit structure determination without the need for direct inter-polymer contacts. The structures described here are the result of pilot studies. We report them because they reveal unique insights into the capabilities of the TELSAM crystallization chaperone.

## 2. Methods

### 2.1. Cloning of the vWa alone

Residues 38–217 from human ANTXR cell adhesion molecule 2 (ANTXR2, also known as capillary morphogenesis gene 2 (CMG2)) (Uniprot: P58335) comprising the von Willebrand domain (vWa) were reverse-translated, codon optimized (DNAworks, http://helixweb.nih.gov/dnaworks, RRID:SCR_008470) [9] and synthesized as a gene fragment (Twist Biosciences). The two cysteines in this region of the gene were first mutated to alanine. The gene fragment was cloned into a custom pET42_SUMO vector using Gibson assembly [10], transformed into BL21(DE3) cells and sequence verified. pET42_SUMO was derived from pET42 by inserting a 10xHis–yeast SMT3–XhoI fragment between its NdeI and AvrII sites, in place of the GST gene (Novagen).

### 2.2. Cloning of 1TEL-flex-vWa

A gene fragment that placed the human ANTXR cell adhesion molecule 2 vWa domain (residues 40–217) [11] after residues 47–124 (the SAM domain) of human ETS variant transcription factor 6 (also known as TEL, Uniprot: P41212) was designed and cloned as described above. The TEL SAM domain (TELSAM) arginine 49 was mutated to alanine to alleviate a potential clash with the vWa domain. Other mutations in this gene relative to the human sequence were valine 112 to alanine and lysine 122 to alanine. A single alanine linker was placed between the TELSAM and the vWa domain, all cysteines were mutated to alanines and vWa arginine 41 was mutated to alanine to alleviate a potential clash with TELSAM. Overlap polymerase chain reaction mutagenesis and Gibson assembly were then used to change the TELSAM alanine 112 to glutamate to make polymer formation triggerable by a reduction in pH, as previously described [5]. This construct was also transformed into BL21(DE3) cells and sequence verified.

### 2.3. Cloning of 3TEL-rigid-DARPin

A gene fragment that placed the sequence of the Designed Ankyrin Repeat Protein (DARPin) from PDB ID 4J7W [12] after three successive TELSAM domains from PDB ID 2QAR [6] was designed and cloned as described above. The flexible linker between the two TELSAM domains from PDB 2QAR was used between each of the three TELSAM domains in this new construct. The rigid linker between the C-terminal TELSAM domain and the T4 lysozyme target protein from PDB ID 2QAR was modified and placed between the C-terminal TELSAM domain and the DARPin in the 3TEL-rigid-DARPin construct.

### 2.4. Protein expression and purification

A stab of frozen cell stock was used to inoculate 40–60 ml of Luria–Bertani (LB) medium supplemented with 0.35% glucose and 100 µg ml^−1^ kanamycin. The following day, 10 ml of the overnight culture was diluted into 1 l of LB medium supplemented with 0.05% glucose and 100 µg ml^−1^ kanamycin. This was again shaken at 37°C and 250 r.p.m. At an optical density of 0.5, isopropyl β-D-1-thiogalactopyranoside (IPTG) was added to a final concentration of 100 µM. The culture was cooled to 18°C and shaken at 250 r.p.m. for an additional 20 h. The cells were collected by centrifugation, snap-frozen in liquid nitrogen and stored at −80°C.

All purification steps were completed on ice or in a 4°C refrigerator. Wet cell paste (5–20 g) was resuspended in a fivefold excess of wash buffer (50 mM Tris, 200 mM KCl, 50 mM imidazole, 10 mM MgCl_2_), supplemented with 1 mM phenylmethylsulfonyl fluoride (PMSF) and 100 µM dithiothreitol (DTT). Lysozyme, deoxyribonuclease I and ribonuclease were added to the resuspended cells to final concentrations of 20 µM, 800 nM and 2 µM, respectively. pH 7.3 was used for the vWa alone while pH 8.8 was used for 1TEL-flex-vWa and 3TEL-rigid-DARPin. The cells were lysed by sonication for 25 cycles of 12 s on/59 s off at 60% power (Qsonica Q500) in a spinning ice bath. Cell suspensions of 3TEL-rigid-DARPin were instead homogenized at 120 MPa for two passes (NanoDeBEE 45–2; BEE International). In each case, the resulting lysate was clarified by centrifugation at 40 000*g* and applied to 2–3 ml of HisPure Ni-NTA resin (Thermo Scientific), which was then washed with 7 column bed volumes (CV) of wash buffer. The protein was then eluted with about 7 CV of elution buffer (50 mM Tris, pH 8.8, 200 mM KCl, 400 mM imidazole, 10 mM MgCl_2_) and desalted using several PD-10 desalting columns in parallel (Cytiva). Typical protein yields per litre of cell culture were 50 mg for the vWa alone, 120 mg for 1TEL-flex-vWa and 60 mg for 3TEL-rigid-DARPin. The SUMO tag was removed by the addition of 2–30 mg of SUMO protease [13] and DTT to 100 μM, and the cleavage reaction was allowed to proceed overnight at 4°C. The SUMO tags and SUMO protease were removed by passing the protein solution over 2 ml of fresh Ni-NTA resin. The protein was then diluted eightfold with water and applied to 4 ml of either CaptoQ or Source 15Q anion exchange resin (Cytiva). The 1TEL-flex-vWa and 3TEL-rigid-DARPin bound to the anion exchange resin and were eluted in a KCl gradient. The vWa domain eluted in the flow through, while contaminants bound to the resin and were thus removed. The protein was further purified by size exclusion chromatography using a 100 ml Superdex 200 preparation grade column (Cytiva). Following size exclusion chromatography (SEC), the proteins were buffer exchanged into 12.5 mM Tris, pH 8.8, 200 mM KCl and 10 mM MgCl_2_. PMSF, phosphoramidon and pepstatin A were added to 1TEL-flex-vWa and 3TEL-rigid-DARPin preparations to final concentrations of 1 mM, 25 µM and 1 µM, respectively. MgCl_2_ was omitted from all steps in preparations of 3TEL-rigid-DARPin.

### 2.5. Crystallization and diffraction of the vWa alone

A 1.2 µl aliquot of 20, 30 or 40 mg ml^−1^ vWa was combined with a 1.2 µl aliquot of reservoir solution in a sitting drop format (SPT Labtech Mosquito) using commercially available crystallization screens (PEG Ion, Index, Salt-Rx, PEG-Rx (Hampton Research)) and custom optimization screens (PEG-custom, Bis-Tris magnesium formate, PEG-potassium thiocyanate, PEG-malonate) were employed. From 35 to 39 days after setting the trays, diamond-shaped plate crystals appeared under various conditions, with the largest (100 × 50 × 10 µm) appearing in 100 mM glycine, pH 9.0–9.5, 28–30% PEG 3350 or in 100 mM KSCN, 100 mM glycine, pH 9.0 and 28–30% PEG 3350. The crystals were mounted using crystallization reservoir solution with 20% glycerol as a cryo-protectant prior to freezing in liquid nitrogen. X-ray diffraction images were collected remotely at SSRL beamline 9–2. These crystals diffracted to around 2.4 Å resolution (1.9–3.1 Å across 18 crystals, *I*/*σ* ≥ 2), were readily indexed in a C-centred monoclinic unit cell with average dimensions *a* = 78 Å *b* = 89 Å, *c* = 60 Å, exhibited low estimated mosaicity (0.3–0.5°) and indexed around 54% of the non-ice reflections (19–95% across 18 crystals).

### 2.6. Crystallization and diffraction of 1TEL-flex-vWa

The protein was crystallized at 1, 2, 5, 10, 15 and 20 mg ml^−1^ as described above. Commercially available crystallization screens (PEG Ion and Index (Hampton Research)) and custom screens (PEG-Tacsimate and PEG-Malonate) were employed. Crystals appeared in 3–10 days under various conditions, with the largest (100 × 100 × 500 µm) in 100 mM Bis-Tris, pH 5.7 and 3.0 M NaCl. The crystals were mounted, frozen and diffracted as described above. These crystals diffracted to around 2.9 Å resolution (2.8–3.0 Å across three crystals, *I*/*σ* ≥ 2), were readily indexed in a primitive hexagonal unit cell with dimensions *a* = *b* = 104 Å, *c* = 57 Å, exhibited low estimated mosaicity (0.3–0.6°) and indexed 59% of the non-ice reflections (44–72% across three crystals).

### 2.7. Crystallization and diffraction of 3TEL-rigid-DARPin

The protein was crystallized at 15 mg ml^−1^ as described above. Commercially available crystallization screens (PEG Ion, SaltRX, PEG-Rx and Index (Hampton Research)) and custom screens (PEG-custom and Bis-Tris magnesium formate) were employed. Thin plate crystals appeared in 3 days under many conditions, with the largest (100 × 100 × 10 µm) in 200 mM L-proline, 100 mM HEPES, pH 7.4, 10% w/v PEG 3350 or in 50 mM MgCl_2_, 100 mM HEPES, pH 7.3, 30% v/v PEG 550 MME. The crystals were mounted, frozen and diffracted as described above. A single thin plate crystal diffracted to 3.2 Å resolution (I/*σ* ≥ 2.0) and was initially indexed into a primitive orthorhombic unit cell with dimensions *a* = 45.9 Å, *b* = 63.6 Å, *c* = 165.6 Å, *α* = *β* = *γ* = 90°. The top indexing solution included only 38% of the non-ice reflections. The P2_1_2_1_2_1_ space group readily gave a clear molecular replacement solution but multiple attempts at subsequent extensive refinement were unable to produce *R*_work_/*R*_free_ values below 0.27/0.31. The data were rescaled into space group P12_1_1 with dimensions *a* = 46.0 Å, *b* = 63.6 Å, *c* = 166.0 Å, *α* = 90°, *β* = 90.162°, *γ* = 90°. This indexing solution also included 38% of the non-ice reflections. Molecular replacement again readily identified a solution. Subsequent refinement using space group P12_1_1 resulted in much lower *R*_work_/*R*_free_ values (0.23/0.24).

### 2.8. Data reduction and structure solution

The datasets were processed using the Autoproc pipeline (https://www.globalphasing.com/autoproc/, RRID:SCR_015748) [14] and the Staraniso algorithm (http://staraniso.globalphasing.org/cgi-bin/staraniso.cgi, RRID:SCR_018362) [15]. The phases were solved by molecular replacement using Phenix (https://www.phenix-online.org/, RRID:SCR_014224) Phaser (https://www.phenix-online.org/documentation/reference/phaser.html, RRID:SCR_014219) [16,17]. The structure then went through alternating stages of rebuilding in Coot [18] and refinement in Phenix Refine (https://www.phenix-online.org/documentation/reference/refinement.html, RRID:SCR_016736) [19,20]. Translation, libration and screw (TLS) parameters were refined as well, using TLS groups found by the TLSMD server [21]. Refinement was assisted using statistics from the MolProbity server (http://molprobity.biochem.duke.edu, RRID:SCR_014226) [22].

## 3. Results

**Figure 2.**
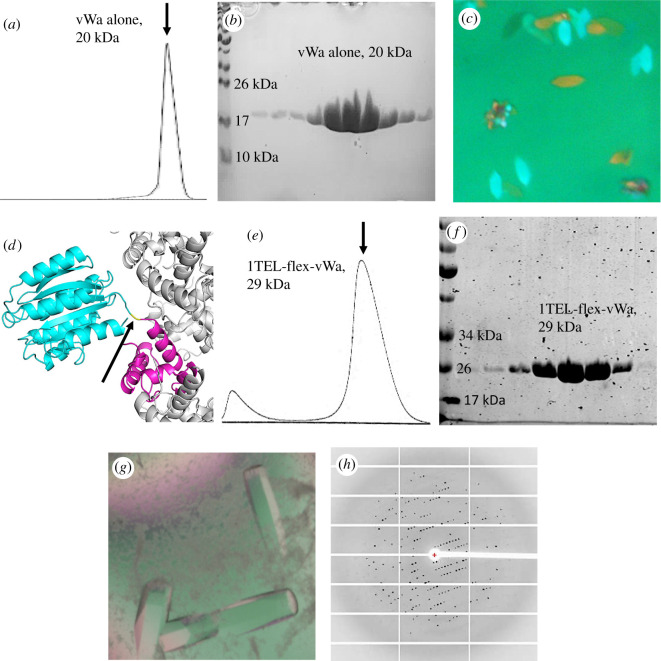
TELSAM accelerates the crystallization rate of a genetically fused vWa domain. (*a*) SEC trace of vWa alone. (*b*) A post-SEC polyacrylamide gel electrophoresis (PAGE) gel of purified vWa alone. (*c*) Representative crystals of vWa alone. Scale bar is 100 μm. (*d*) Design model of a 1TEL-flex-vWa fusion in cartoon representation, with TELSAM in magenta and the vWa in cyan. Other subunits of the TELSAM polymer are shown in white. The linker is coloured yellow and indicated with an arrow. (*e*) SEC trace of 1TEL-flex-vWa. (*f*) A post-SEC PAGE gel of purified 1TEL-flex-vWa. (*g*) Crystals of 1TEL-flex-vWa. Scale bar is 100 μm. (*h*) Representative diffraction pattern from a crystal of 1TEL-flex-vWa.

Fusion to TELSAM increases the crystallization rate and propensity of the human ANTXR cell adhesion molecule 2 von Willebrand factor type A (vWa) domain. The vWa domain was chosen because it has been successfully crystallized previously, has a known structure and is only moderately soluble. These properties make the vWa domain an excellent representative target protein to evaluate potential crystallization chaperones. As this was a pilot study, we initially evaluated TELSAM with target proteins that we reasonably expected would crystallize. We sought to quantitatively evaluate whether fusion to TELSAM could enhance the properties of vWa domain crystallization relative to the vWa alone. We produced pure, soluble, monodisperse vWa domain (figure 2*a,b*) and executed crystallization trials at a range of protein concentrations and using a range of commercially available and custom-made crystallization screens. From 35 to 39 days after setting the trays, thin plate-like crystals appeared in four distinct crystallization conditions (figure 2*c*). These crystals diffracted to an average resolution of 2.4 Å (table 1).

**Table 1.** Crystallization time, propensity and diffraction quality of vWa constructs.

construct	days to crystal appearance	number of commercial conditions with crystals	number of commercial conditions screened	average size of largest crystals (long axis, μm)	average diffraction resolution of crystals (Å)	average fraction of non-ice reflections indexed (%)
vWa alone	35–39	4 (1%)	384	100	2.4	54
1TEL-flex-vWa	3–10	9 (3%)	288	400	2.9	59

We next modelled the shortest flexible genetic fusion of the vWa domain [11] to the C-terminus of a single TELSAM monomer (1TEL, PDB ID: 1JI7) [5] using PyMOL (Schrödinger, http://www.pymol.org/, RRID:SCR_000305) and Foldit (http://fold.it/, RRID:SCR_003788) [23]. We determined that the vWa domain could be flexibly fused to TELSAM with a linker consisting of a single alanine residue (figure 2*d*). This construct included the critical TELSAM V112E mutation that makes TELSAM polymerization pH dependent [5]. The resulting protein was produced, purified and crystallized in a manner identical to the solitary vWa domain above (figure 2*e*,*f*), except at pH 8.8 and adding a cocktail of protease inhibitors to the pure protein immediately before setting crystallization drops (to prevent cleavage of the TELSAM–vWa linker by trace proteases). Crystals of 1TEL-flex-vWa appeared in 3–10 days in nine distinct crystallization conditions (figure 2*g*), with the largest of these diffracting to an average resolution of 2.9 Å (figure 2*h* and table 1). Notably, crystals of 1TEL-flex-vWa could be obtained with protein concentrations as low as 1 mg ml^−1^ in the crystallization drops (at a 1 : 1 protein : reservoir ratio). Concentrations of 1, 2, 5, 10, 15 and 20 mg ml^−1^ were tested. While crystals of 1TEL-flex-vWa appeared in as little as 3 days at 20 mg ml^−1^, they required 10 days to appear at 1 mg ml^−1^. This suggests that fusion to TELSAM could allow protein crystallization at relatively low protein concentrations, thus facilitating crystal formation in cases where the amount or solubility of the target protein is limited. 1TEL-flex-vWa could be successfully produced and crystallized by three independent teams of students in our research group. The crystallization time and propensity and crystal quality of these constructs are summarized in table 1 and table 2.

**Table 2.** Crystallographic data collection and refinement statistics. Statistics for the highest resolution shell are shown in parentheses. CC, correlation coefficient.

construct	1TEL-flex-vWa	3TEL-rigid-DARPin
PDB ID	7N1O	7N2B
data collection		
X-ray source	SSRL 9–2	SSRL 9–2
wavelength	0.979460	0.979460
detector type	Pilatus 6M PAD	Pilatus 6M PAD
detector distance (mm)	350	350
resolution range	38.16–2.77 (2.869–2.77)	40.16–3.221 (3.336–3.221)
space group	P 6_5_	P 1 2_1_ 1
cell a, b, c (Å)	103.396, 103.396, 56.55	45.962, 63.625, 166.005
cell *α*, *β*, *γ* (°)	90, 90, 120	90, 90.162, 90
total reflections	47 554 (5106)	46 124 (4911)
unique reflections	8679 (878)	13 842 (1491)
multiplicity	5.5 (5.8)	3.3 (3.3)
completeness (%)	97.10 (97.99)	87.26 (95.36)
*I*/*σI*	18.35 (2.88)	10.98 (2.46)
Wilson B-factor	71.05	84.40
R-merge	0.05193 (0.6253)	0.07087 (0.5057)
R-meas	0.05753 (0.6876)	0.08449 (0.6052)
R-pim	0.024 (0.2779)	0.04539 (0.3275)
CC1/2	0.999 (0.899)	0.999 (0.906)
CC*	1 (0.973)	1 (0.975)
refinement		
reflections used in refinement	8667 (878)	13 776 (1480)
reflections used for R-free	432 (44)	681 (69)
R-work	0.2030 (0.3449)	0.2262 (0.3233)
R-free	0.2285 (0.3904)	0.2425 (0.3735)
CC (work)	0.965 (0.802)	0.956 (0.849)
CC (free)	0.942 (0.519)	0.969 (0.720)
number of non-H atoms	1932	6067
macromolecule atoms	1921	6067
ligands	3	0
water	8	0
protein residues	257	806
bond length RMS deviations	0.009	0.003
bond angle RMS deviations	0.86	0.56
Ramachandran stats (%)		
favoured (%)	98.04	98.36
allowed (%)	1.96	1.64
outliers (%)	0.00	0.00
rotamer outliers (%)	0.00	0.00
clashscore	8.23	4.73
B-factors (Å2)		
average B-factor	75.23	92.51
macromolecules	75.26	92.51
ligands	92.60	N/A
water	61.89	N/A
number of TLS groups	4	6

**Figure 3.**
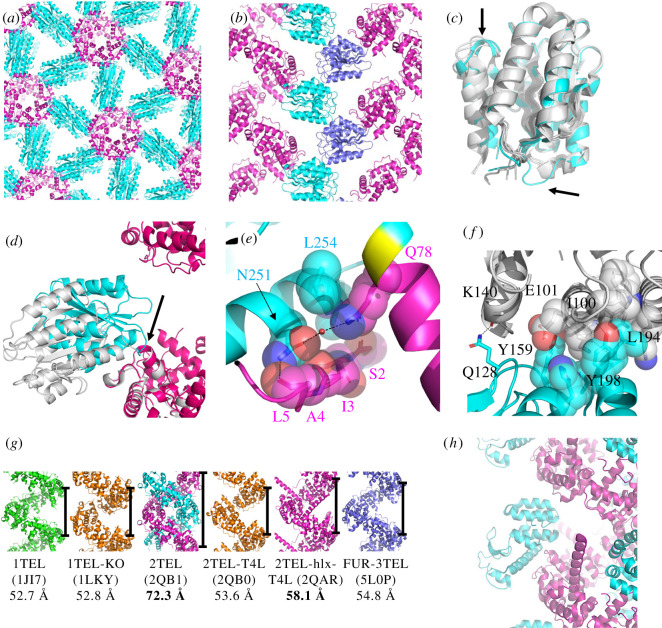
Detail of the 1TEL-flex-vWa crystal structure and lattice. (*a*) Crystal lattice of 1TEL-flex-vWa, in cartoon representation with TELSAM in magenta and the vWa in cyan. A black outline denotes each sub-domain of a single polypeptide. (*b*) Side view of the 1TEL-flex-vWa crystal lattice, showing two TELSAM polymers (magenta) and selected vWA domains (cyan and purple). (*c*) Superposition of the vWa domain from 1TEL-flex-vWa (cyan) onto previously published vWa structures (grey, PDB IDs: 1SHU, 1SHT [11], 1T6B [24] and 1TZN [25]). Significant differences from previous structures are indicated with arrows. (*d*) Comparison of the design model (white) and crystal structure (magenta and cyan) of 1TEL-flex-vWa (the region of the linker that becomes α-helical is indicated with an arrow). Other copies of the vWa domain have been omitted for clarity. (*e*) Detail of the *cis* interface between 1TEL (magenta) and vWa (cyan). Hydrogen bonds are shown as black dashes. The single alanine linker is shown in yellow. (*f*) Detail of the *trans* interface between two vWa units, coloured cyan and grey. (*g*) Comparison of the helical rise from previously published TELSAM crystal structures [5–8]. The relative rise of a single turn of each helix is denoted with a black bar. Fused target proteins have been omitted. (*h*) Detail of T4 lysozyme (cyan) intercalation into the TELSAM polymer helix (magenta) of PDB ID: 2QAR [6].

Molecular replacement with the 1TEL-flex-vWa datasets was carried out by separately placing the structures of 1TEL (PDB ID: 2QB1) [6] and the vWa domain (PDB ID: 1SHU) [11]. We determined the space group to be P6_5_ (as expected owing to the sixfold symmetric, left-handed helical nature of the 1TEL polymer), with one molecule of 1TEL-flex-vWa per asymmetric unit. In the 1TEL-flex-vWa crystallographic lattice, the vWa domains make a head-to-head interaction, leaving considerable aqueous space (59.6% solvent content). Two molecules of the vWa domain separate adjacent TELSAM polymers, and there are no direct inter-TELSAM polymer contacts, a feature not previously observed in crystal structures of TELSAM–target protein fusions (figure 3*a*,*b*). Despite the lack of direct inter-polymer contacts and the high degree of aqueous space, crystals of 1TEL-flex-vWa exhibit minimal anisotropy, with the anisotropic diffraction limits of the unit cell axes being *a* = *b* = 2.60 Å, *c* = 2.55 Å [15] and moderate disorder, with 59% of reflections contributing to the top indexing solution.

The vWa from the crystal structure of 1TEL-flex-vWa differs from previously published structures of the vWa [11,24,25] only in the conformations of two of its surface loops (figure 3*c*). The average RMSD between the vWa domain of 1TEL-flex-vWa and each of the four published vWa structures is 0.482 Å, while the average RMSD of all possible pairings of the four published vWa structures is 0.431 Å. In the 1TEL-flex-vWa structure, the vWa unit adopts a position different from that of the design model, packing against the TELSAM polymer. The single amino acid alanine linker becomes an extension of the 1TEL C-terminal α-helix (figure 3*d*). The C-terminal α-helix of the vWa domain packs against the N-terminus of 1TEL, burying 476 Å^2^ of solvent-accessible surface area (average of both sides of the interface) but making minimal interactions. Specifically, the vWa Leu 254 side chain makes van der Waals interactions to the 1TEL Gln 78 side chain. The vWa Asn 251 side chain amide carbonyl makes a direct hydrogen bond to the 1TEL Leu 5 main chain carbonyl and a water-mediated hydrogen bond to the 1TEL Gln 78 side chain amide nitrogen. The vWa Asn 251 side chain makes additional van der Waals contacts to the main chain of 1TEL Ile 3 and the main chain and side chain of Ala 4 (Figure 3*e*). These interactions confirm that flexibly fused target proteins can find consistent binding modes against the TELSAM polymer when presented with the fast interaction on-rate conferred by covalent attachment.

The inter-vWa interface buries 491 Å^2^ of solvent-accessible surface area and is largely hydrophobic, with minimal polar interactions. Leu 194 from a vWa molecule packs into a hydrophobic pocket formed by the Trp 99, Ile 100, Tyr 103 and Lys 150 of a second vWa molecule. Additionally, Gln 128 and Tyr 198 from the first vWa molecule, respectively, make hydrogen bonds to the backbone carbonyl oxygen of Lys 240 and the backbone amide nitrogen of Ile 100 from the second vWa molecule. Finally, Tyr 159 from the first vWa molecule makes an anion–π interaction with the side chain carboxylate of Glu 101 and a cation–π interaction with the side chain carbonyl oxygen of Gln 104, both from the second vWa molecule. Conversely, Asn 104 and the Ile 100 from this second vWa molecule pack into a largely hydrophobic pocket formed by the His 161, Leu 194, Val 195 and Tyr 198 of the first vWa molecule. Asn 104 additionally makes a hydrogen bond to the backbone amide nitrogen of Leu 194 (figure 3*f*). This specific inter-vWa contact has not been observed in any previously reported structure of the vWa domain [11,24,25]. In view of the fact that a flexibly fused target protein can find a rigid conformation relative to the TELSAM polymer by packing against the polymer, the rigid hydrophobic connection between vWa domains suggests that, while direct inter-polymer contacts are dispensable in forming well-diffracting crystals, a rigid transform between adjacent TELSAM polymers is still required.

Among previously reported TELSAM crystal structures, the TELSAM polymer helix has an average helical rise of 53.5 ± 0.7 Å (figure 3*g*) [5–8]. Notable exceptions to this include the structure of 2TEL alone, which forms a double helix and so has a greatly expanded helical rise of 72.3 Å to accommodate the second intercalated helix [6]. Another exception is the structure of 2TEL fused to T4 lysozyme via a long α-helix, which has a slightly expanded helical rise of 58.1 Å, apparently because of partial intercalation of the lysozyme unit into the polymer helix (figure 3*h*) [6]. The new structure of 1TEL-flex-vWa likewise has a slightly expanded helical rise of 56.5 Å. We rule out the possibility that the vWa domain perturbs the 1TEL helical rise via intercalation into the 1TEL polymer helix because the vWa domains make no contacts to the next turn of their host 1TEL polymers (the nearest resolved atoms of each vWa are at least 7.9 Å from the nearest atoms of the next turn of its host 1TEL polymer; figure 3*b*). A more likely explanation is that this degree of helical rise has been dictated by the spacing required to achieve the observed inter-vWa crystal contacts. The fact that the 1TEL helical rise is perturbed suggests that TELSAM may adjust its helical rise to accommodate the crystal packing interactions of fused target proteins. The 1TEL-flex-vWa structure also provides further evidence that the flexibility in the TELSAM helical rise is not necessarily a detriment to the growth of ordered crystals.

**Figure 4.**
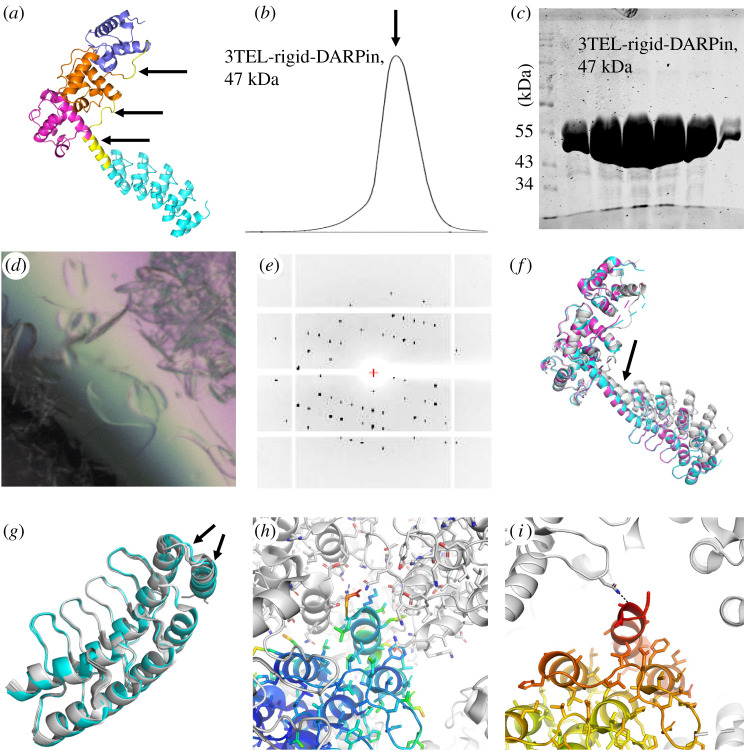
Production and detail of the 3TEL-rigid-DARPin crystal structure. (*a*) Design model of 3TEL-rigid-DARPin, with successive 1TEL domains shown in purple, orange and magenta and the DARPin in cyan. Linkers are coloured yellow and indicated with arrows. (*b*) SEC trace of 3TEL-rigid-DARPin. (*c*) A post-SEC PAGE gel of 3TEL-rigid-DARPin. (*d*) Representative crystals of 3TEL-rigid-DARPin. Scale bar is 100 μm. (*e*) Representative diffraction pattern of 3TEL-rigid-DARPin. (*f*) Comparison of the design model (magenta) with the crystal structure (cyan and magenta) of 3TEL-rigid-DARPin, with an arrow to indicate the shift of the DARPin and connecting α-helix. (*g*) Superposition of the DARPin domains from the 3TEL-rigid-DARPin crystal structure (cyan) and from the published structure of this same DARPin (white, PDB ID: 4J7W) [12]. Significant differences from the previous structure are indicated with arrows. (*h*) Detail of the crystal packing from the previous crystal structure of the DARPin domain. One of the DARPin domains has been coloured according to the crystallographic B-factor. (*i*) Detail of the crystal contacts from the crystal structure of 3TEL-rigid-DARPin. One of the 3TEL-rigid-DARPin units has been coloured according to the crystallographic B-factor. The view angle is the same as in (*h*).

In a related pilot study, we explored whether TELSAM could scaffold a DARPin for potential use with conformationally heterogeneous target proteins. While the DARPin is only 17 kDa and approximately 57 × 29 × 21 Å in size, a potential target protein bound to the DARPin could be much larger. We thus chose 3TEL to avoid steric hinderance between neighbouring copies of such a target protein around the TELSAM polymer axis. We modelled a rigid α-helical fusion between the C-terminal α-helix of 3TEL and the N-terminal α-helix of a DARPin (PDB ID: 4J7 W) [12] using PyMOL (Schrödinger). We chose a DARPin orientation that would allow it to non-covalently bind a second target protein using the DARPin's canonical binding surface while minimizing clashes between that second target protein and the TELSAM polymer (figure 4*a*). The 3TEL construct was designed as described above. The resulting protein was produced and crystallized in a manner identical to the 1TEL-flex-vWa construct above (figure 4*b*,*c*), except that Mg^2+^ was omitted. Thin plate crystals appeared in 3 days under various conditions, diffracting to around 3.2 Å resolution (figure 4*d*,*e*).

Molecular replacement into the 3TEL-rigid-DARPin dataset was carried out by separately placing models of 3TEL and the DARPin (PDB ID: 4J7W) [12]. We determined the space group to be P12_1_1, consistent with the twofold symmetric, left-handed helical nature of the 3TEL polymer, with two molecules of 3TEL-rigid-DARPin per asymmetric unit. The 3TEL-rigid-DARPin crystal structure differs from the design model only in that the DARPin translates as much as 12.5 Å in the crystal structure relative to the 3TEL unit, relative to the design model (figure 4*f*). This confirms that an α-helical connection retains some residual flexibility, a feature observed in other studies using rigid α-helical fusions [26–29]. This residual flexibility may have allowed the DARPin to access productive crystal packing arrangements.

The DARPins from the crystal structure of 3TEL-rigid-DARPin differ from the previously published structure of this same DARPin [12] only in the conformations of the C-terminal α-helix and the surface loop preceding it (figure 4*g*). The average RMSD between the DARPin domains in the 3TEL-rigid-DARPin structure and those in the previous DARPin structure is 0.404 Å. The RMSD between the individual DARPin domains in the 3TEL-rigid-DARPin structure is 0.249 Å, while the average RMSD between the chains in the previous DARPin structure is 0.407 Å. Comparison of the crystal packing in the previous DARPin structure and the 3TEL-rigid-DARPin structure reveals that the C-terminal α-helix of the DARPin in the previous structure is more tightly packed (38.1% solvent content) and makes many more crystal contacts than the same α-helix in the 3TEL-rigid-DARPin structure (52.6% solvent content; figure 4*h*,*i*). The lack of extensive crystal contacts made by the C-terminal α-helix in the 3TEL-rigid-DARPin structure and the observation that this α-helix is the most disordered region of the structure (has the highest refined B-factors) might explain the differences in the helix and loop conformations relative to the original DARPin structure. This observation suggests that fusion to TELSAM may allow visualization of more dynamic protein conformations than can be seen in more tightly packed crystal lattices.

**Figure 5.**
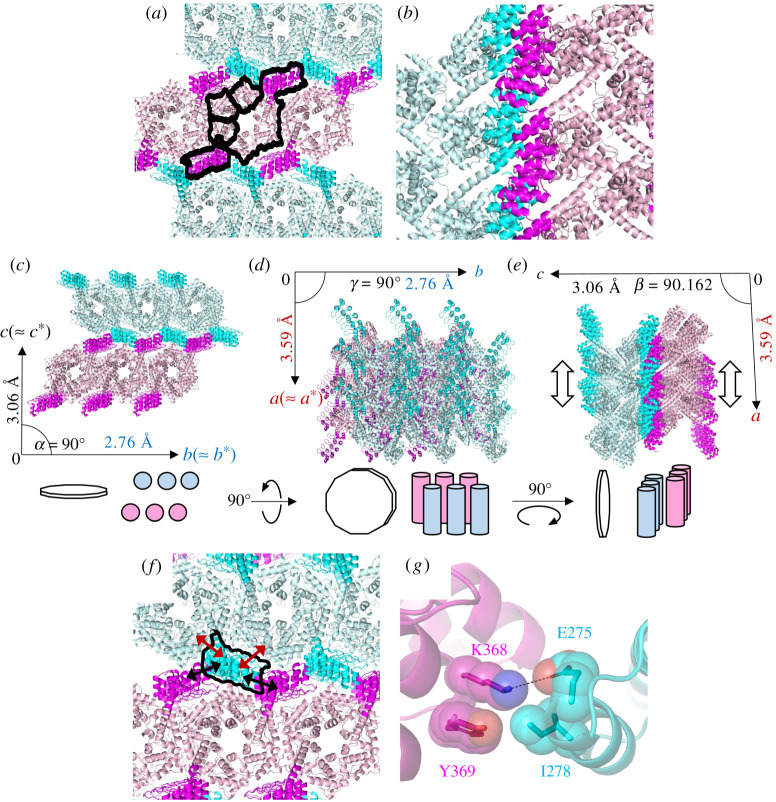
Additional features of the 3TEL-rigid-DARPin crystal structure. (*a*) Crystal lattice of 3TEL-rigid-DARPin. Black outlines highlight a single polymer as well as the domains of a single polypeptide subunit within that polymer. (*b*) Side view of the 3TEL-rigid-DARPin crystal lattice, showing two TELSAM polymers (light blue and light pink) and selected DARPin domains (cyan and magenta). (*c–e*). Diagram relating the 3TEL-rigid-DARPin crystal packing to the crystallographic unit cell, shown from three view angles. Two polymer layers are shown, with three polymers in each layer and 6 × 3TEL-rigid-DARPin subunits in each polymer (three turns of each polymer). The unit cell axes are denoted with labelled arrows and approximately correspond to the reciprocal space axes and the axes of an ellipsoid approximating the anisotropic diffraction limits. In each view angle, the origin lies in the plane of the page while the unseen unit cell axis projects out of the page towards the reader. These anisotropic diffraction limits approximately correspond to each of the unit cell vectors and appear in coloured type. Schematics are given below each view angle to indicate the orientation of both the TELSAM polymers (pink and blue) and the crystal as a whole (white prismatic disc) in each view. The predicted vertical displacement of the two polymer sheets relative to one another is indicated with thick white arrows in (*e*). (*f*) Schematic of crystal contacts made by a single DARPin molecule, highlighted with a black line. Contacts to its own 3TEL polymer layer are indicated with red arrows, while contacts to DARPins from an adjacent polymer layer are indicated with black arrows. (*g*) Interface between a DARPin (cyan) and another DARPin (magenta) from an apposed 3TEL polymer layer, with selected amino acid side chains shown as sticks and transparent spheres and a salt bridge shown as a black dash. This is the same interface indicated with black arrows in (*f*).

In the 3TEL-rigid-DARPin crystallographic lattice, 3TEL polymers pack side by side in lateral layers (figure 5*a*). All of the 3TEL polymers in a given layer are oriented in the same N → C direction but are oriented oppositely from the 3TEL polymers in the preceding or following layers (figure 5*b*). This is the first reported instance of a crystal structure in which TELSAM polymers are not all oriented in the same N → C direction. Successive 3TEL layers are separated by a layer of DARPins that alternate emanating from one or the other of the layers (figure 5*a*). While the crystal packing of the vWa units slightly expanded the helical rise of the associated 1TEL polymer (figure 3*b*), the DARPin–DARPin crystal packing had a profound impact on the rise of the 3TEL polymer, reducing it to 45.9 Å, the minimum helical rise reported to date for a TELSAM polymer (figures 3*g* and 5*b*). The DARPin units do not intercalate into the 3TEL polymer, ruling out target protein intercalation as a cause of the reduced degree of 3TEL helical rise and further suggesting that inter-target crystal packing may dictate the rise of the TELSAM helical polymer.

Unlike the minimally anisotropic diffraction of 1TEL-flex-vWa crystals, 3TEL-rigid-DARPin crystals exhibited significant anisotropy as estimated using the Staraniso software [15]. In the case of 3TEL-rigid-DARPin crystals, an ellipsoid approximating the diffraction limits of statistically significant reflection intensities in reciprocal space approximately aligns with the a*, b* and c* axes (ellipsoid axis *a* = 0.983a* – 0.182c*, axis *b* = b*, axis *c* = 0.013a* + c*). While the 3TEL-rigid-DARPin crystals were better refined in space group P12_1_1, the unit cell *β* angle is 90.162° (*α* = *γ* = 90°), meaning that the unit cell symmetry is very close to an orthorhombic unit cell. Indeed, as there are two copies of 3TEL-rigid-DARPin per asymmetric unit, the diffraction data were readily scaled into space group P2_1_2_1_2_1_, although refinement could not produce a model with *R*_work_/*R*_free_ values below 0.27/0.31 in that space group. Since the P12_1_1 unit cell of 3TEL-rigid-DARPin crystals is very nearly an orthorhombic P2_1_2_1_2_1_ unit cell, the reciprocal space axes approximately map onto the real space axes of the unit cell (a* = *a*, b* ≈ *b*, c* = *c*). The anisotropic diffraction limits of the 3TEL-rigid-DARPin unit cell along each real space unit cell axis were thus approximately *a* = 3.59 Å, *b* = 2.76 Å, *c* = 3.06 Å (figures 5*c–e*).

The anisotropy along each unit cell axis can be correlated with the internal arrangement of the unit cell and with the overall shape of the 3TEL-rigid-DARPin crystal (figures 5*c–e*). When correlated with the unit cell contents, these anisotropic diffraction limits suggest that the largest atomic displacement error lies in the vertical register between the helical 3TEL polymers (parallel to the helical axes of the polymers, along unit cell axis *a*), either within each 3TEL polymer layer or between successive layers. As the 3TEL polymers pack tightly against their neighbours within each polymer layer, we propose that this displacement error most likely lies in the vertical register between successive 3TEL polymer layers such that successive polymer layers are able to shift vertically relative to each other in the crystal (moving parallel to the helical axes of the individual polymers) (figure 5*e*). The gear-like intercalation of DARPins from adjacent 3TEL polymer layers may have limited the horizontal displacement of the polymer layers relative to each other (in the plane of a single polymer layer but perpendicular to the helical axis of the polymers, along unit cell axis *b*; figure 5*c*).

Analysis of the crystal position during diffraction image collection reveals that the 3TEL polymer layers lie parallel to the long axes of the thin plate crystal. The thin/short axis of the crystal lies perpendicular to the planes of the 3TEL polymer layers (figure 5*c–e*) and suggests that 3TEL-rigid-DARPin crystals may experience a significant growth defect in this dimension. Concomitantly, diffraction images collected at angles closer to parallel to the *c*-axis of the unit cell had higher scale factors during data processing. That the *c*-axis of the unit cell lies parallel to the thin/short axis of the crystal may explain the poorer resolution diffraction limits and higher scale factors of reflections collected from this angle but cannot explain the anisotropy in the diffraction limits along the *a* and *b* axes of the unit cell (figure 5*c–e*).

3TEL-rigid-DARPin crystals exhibit minimal crystal contacts between adjacent layers of 3TEL-rigid-DARPin polymers. While a given DARPin makes a fair number of van der Waals and salt bridge contacts to its own host 3TEL polymer (burying 231 Å of solvent-accessible surface area) and to an adjacent 3TEL polymer from the same polymer layer (burying 431 Å of solvent-accessible surface area), it makes no contacts to the 3TEL polymers of adjacent layers. All inter-layer contacts occur solely between DARPins from adjacent polymer layers (figure 5*f*). As they are the only contacts between adjacent 3TEL polymer layers, we were struck by how minimal the inter-DARPin contacts were. Inter-DARPin contacts bury only 147 Å^2^ of solvent-accessible surface area and involve a single salt bridge between Lys 368 on the canonical binding surface of one DARPin and Glu 275 on the back side of a second DARPin. Tyr 369 appears to stabilize the position of Lys 368. Ile 278 from the back side of the second DARPin also makes minimal van der Waals interactions to the Lys 368. (Figure 5*g*). Since this is a DARPin–DARPin contact, each DARPin makes two such contacts to other DARPins from the adjacent polymer layer. For a typical circular plate crystal 100 μm in diameter, this corresponds to approximately 5.4 × 10^8^ such contacts between each pair of neighbouring 3TEL polymer layers. The lateral shifting of 3TEL polymer layers relative to each other may be a consequence of these extremely minimal inter-layer (inter-DARPin) crystal contacts (figure 5*c–g*). The weak inter-DARPin crystal contacts and vertical displacement of polymer layers may also explain the observed diffraction limits, growth defect and scale factor of the thin/short axis of the crystal. Taken together, these observations suggest that the avidity of the inter-layer, inter-DARPin contacts was sufficient to stabilize these otherwise weak interactions.

## 4. Discussion

In comparing the crystallization rate of the vWa domain from human ANTXR2/CMG2 with and without fusion to 1TEL, we provide an example wherein a TELSAM–target protein fusion crystallized more rapidly and with more propensity than the same target protein alone. Testing an expanded set of target proteins with and without fusion to TELSAM will reveal how generalizable this phenomenon is. In particular, target proteins that have proven recalcitrant to crystallization will be tested. We also discovered that TELSAM–target protein fusions can be crystallized at protein concentrations as low as 1 mg ml^−1^. This startling observation is at odds with traditional protein crystallography practices, which require protein concentrations of 5–50 mg ml^−1^ to achieve crystallization. This observation suggests that fusion to TELSAM may allow the crystallization and structure determination of proteins that can only be produced in microgram quantities or that have limited solubility.

We were surprised to discover that the TELSAM polymers themselves need not directly contact one another to form a diffraction-quality crystal lattice. This is a profound discovery because removal of the requirement for adjacent TELSAM polymers to make direct contact significantly extends the theoretical upper size limit of target proteins that can be crystallized using TELSAM. This result also suggests that 1TEL (with six copies of the target protein per helical turn) is a viable crystallization chaperone candidate.

We observed that a flexible linker between the 1TEL subunit and the vWa domain did not abrogate crystal formation or impair diffraction and structure determination, probably because the vWa domain adopted a rigid binding mode against its own 1TEL polymer. The ability to use flexible linkers is compelling because it promises the possibility of designing TELSAM–target fusions based only on the predicted secondary structures of target proteins for which the tertiary structure is unknown. Future work is needed to define the maximum flexible linker length and optimal linker composition that reliably allow the formation of well-ordered crystals using TELSAM.

We have observed further examples of the TELSAM polymer adjusting its helical rise to allow inter-target protein crystal contacts sufficient to form diffracting crystals and report a new minimum helical rise for a single-helix TELSAM polymer of 46.9 Å. Further work is needed to determine whether rigidifying the helical rise of TELSAM is enabling or detrimental to the formation of ordered crystals and whether such is target protein dependent.

We observed that the 3TEL-rigid-DARPin crystal was organized into layers of 3TEL polymers and that adjacent layers made contact through many copies (approximately 5.4 × 10^8^) of a very minimal crystal contact. These observations support the idea that fusion to TELSAM polymers increases the avidity of fused target proteins to stabilize otherwise weak inter-target protein crystal contacts. We propose that this is the principal manner in which TELSAM may increase the crystallization rate and propensity of fused target proteins. Based on the extremely weak individual inter-layer crystal contacts observed in the 3TEL-rigid-DARPin structure, theoretically any monomeric protein that is sufficiently homogeneous in composition and conformation could be crystallized through fusion to TELSAM. This observation calls for continued investigation into the principles and requirements for using TELSAM.

Future studies are underway to determine how generally useful fusion to TELSAM is for generating well-ordered crystals of a greater variety of target proteins, the optimal number of target proteins per turn of the TELSAM helical polymer and whether this optimal number is dependent on the specific target protein. Future work is also needed to determine whether 1TEL, 2TEL and 3TEL each work better with rigid or flexible linkers, whether there is a complex relationship between the optimal number of target proteins displayed per helical turn and the linker type and whether the optimal combination is specific for each target protein.

The TELSAM-mediated structures reported here did not arise from diffraction data extending beyond 2.77 Å, in contrast with previous structures of these target proteins crystallized on their own. The 1TEL-flex-vWa structure reached 2.77 Å resolution, while previous structures of the vWa alone reached 1.81 and 1.5 Å resolution [11]. In our hands, crystals of the vWa alone had an average diffraction limit of 2.38 Å (across 18 well-diffracting crystals), suggesting that some of the resolution loss we observed could be due to operator skill level. Likewise, the 3TEL-rigid-DARPin structure reached 3.22 Å resolution, while the previous structure of this same DARPin sequence attained 1.60 Å resolution [12]. Previously reported crystal structures of TELSAM–target protein fusions likewise did not exhibit resolution beyond 2.30 Å [6,7]. It is possible that the increased solvent content seen in these TELSAM–target fusion crystals (59.6% for 1TEL-flex-vWa and 52.6% for 3TEL-rigid-DARPin) prevented superior resolution. As the average solvent of protein crystals is close to 50% [30], the low resolution of crystals of the 3TEL-rigid-DARPin construct may be explained by other factors as well, such as their degree of disorder. Further work is needed to determine whether fusion to TELSAM imposes a limit on the resolution of resulting diffraction datasets and whether that limit can be extended. We note that synchrotron radiation (SSRL beamline 9–2), a Pilatus 6M pixel array detector (Dectris) and the Autoproc software pipeline [14] were instrumental in mitigating the disorder in the 3TEL-rigid-DARPin crystals and extending the resolution of each of the structures reported here. Based on current evidence, TELSAM may become an appropriate tool to enable the crystallization of recalcitrant proteins, but not for increasing the diffraction resolution of proteins that can already be crystallized on their own.

### 4.1. Opening Up

Integral membrane proteins are often difficult to crystallize because of limited crystal contacts. TELSAM's ability to stabilize weak crystal contacts and to preorder target proteins along its polymer helix may be able to overcome these obstacles. TELSAM's potential to crystallize fused membrane proteins is further supported by previous work showing that TELSAM crystallization was not impaired by even high concentrations of various detergents [6]. Protein complexes are likewise often difficult to crystallize owing to their oligomeric and conformational flexibility. If the problem of premature polymerization (observed when fusing two oligomeric components together) [7,31] can be overcome, TELSAM may be suitable for the crystallization of protein complexes. TELSAM's ability to form crystals with increased aqueous space may allow fused target protein complexes to crystallize using fewer crystal contacts, requiring fewer low-entropy regions on the surfaces of protein complexes. To date, TELSAM has not been shown to directly reduce the conformational flexibility of target proteins. For this task, selectable binding domains, such as DARPins or *α*Reps [32,33], may be more appropriate, although a selected binding domain could additionally be fused to TELSAM if the binding domain is not sufficient to crystallize the bound target protein on its own.

## Data Availability

The coordinates and structure factors of the X-ray crystal structures reported in this study have been deposited in the Worldwide Protein Data Bank under PDB IDs: 7N1O and 7N2B. The amino acid sequences of the constructs are provided in the electronic supplementary material [34].
